# Direct conversion of osteosarcoma to adipocytes by targeting TNIK

**DOI:** 10.1172/jci.insight.137245

**Published:** 2021-02-08

**Authors:** Toru Hirozane, Mari Masuda, Teppei Sugano, Tetsuya Sekita, Naoko Goto, Toru Aoyama, Takato Sakagami, Yuko Uno, Hideki Moriyama, Masaaki Sawa, Naofumi Asano, Masaya Nakamura, Morio Matsumoto, Robert Nakayama, Tadashi Kondo, Akira Kawai, Eisuke Kobayashi, Tesshi Yamada

**Affiliations:** 1 Division of Cellular Signaling, National Cancer Center Research Institute, Tokyo, Japan.; 2 Department of Orthopedic Surgery, Keio University School of Medicine, Tokyo, Japan.; 3 Department of Pulmonary Medicine and Oncology, Graduate School of Medicine, Nippon Medical School, Tokyo, Japan.; 4 Keio University School of Medicine, Tokyo, Japan.; 5 Carna Biosciences Inc., Kobe, Japan.; 6 Division of Rare Cancer Research, National Cancer Center Research Institute, Tokyo, Japan.; 7 Division of Musculoskeletal Oncology, National Cancer Center Hospital, Tokyo, Japan.; 8 Department of Gastrointestinal and Pediatric Surgery, Tokyo Medical University, Tokyo, Japan.

**Keywords:** Cell Biology, Therapeutics, Adult stem cells, Drug therapy, Oncogenes

## Abstract

Osteosarcoma (OS) is an aggressive mesenchymal tumor for which no molecularly targeted therapies are available. We have previously identified TRAF2- and NCK-interacting protein kinase (TNIK) as an essential factor for the transactivation of Wnt signal target genes and shown that its inhibition leads to eradication of colorectal cancer stem cells. The involvement of Wnt signaling in the pathogenesis of OS has been implicated. The aim of the present study was to examine the potential of TNIK as a therapeutic target in OS. RNA interference or pharmacological inhibition of TNIK suppressed the proliferation of OS cells. Transcriptome analysis suggested that a small-molecule inhibitor of TNIK upregulated the expression of genes involved in OS cell metabolism and downregulated transcription factors essential for maintaining the stem cell phenotype. Metabolome analysis revealed that this TNIK inhibitor redirected the metabolic network from carbon flux toward lipid accumulation in OS cells. Using in vitro and in vivo OS models, we confirmed that TNIK inhibition abrogated the OS stem cell phenotype, simultaneously driving conversion of OS cells to adipocyte-like cells through induction of PPARγ. In relation to potential therapeutic targeting in clinical practice, TNIK was confirmed to be in an active state in OS cell lines and clinical specimens. From these findings, we conclude that TNIK is applicable as a potential target for treatment of OS, affecting cell fate determination.

## Introduction

Osteosarcoma (OS) is a rare malignant bone tumor that mainly affects adolescents and young adults. The outcome of patients with localized OS has improved significantly, with recent advances in multidisciplinary patient management. However, the 5-year survival rate of patients with OS with distant metastasis is still around 30% (1), and this figure has remained unchanged over the last few decades (2, 3). Because of the genomic complexity and heterogeneity of OS (4, 5), no form of molecular therapy has been approved (6–8), and conventional chemotherapy still remains the mainstay. Furthermore, it has become apparent that immunotherapy is not as effective for OS as it is for other malignancies. In a recent phase II clinical trial, only 1 of 22 patients with OS (5%) treated with the immune checkpoint inhibitor pembrolizumab achieved a partial response (9). Therefore, development of a fresh therapeutic approach would seem desirable. The involvement of Wnt signaling in the pathogenesis of OS has been implicated. OS is believed to be derived from mesenchymal stem cells (MSCs), which have the potential to differentiate into multiple cell lineages, including osteoblasts and adipocytes (10). Wnt signaling has been shown to commit MSCs to osteoblastogenesis (11), but sustained activation would likely prevent proper differentiation and give rise to OS progenitors (12, 13). It has been reported that inhibition of Wnt signaling converts the fate of mesenchymal precursors from osteoblasts to adipocytes through switching of lineage-specific transcription factors (11, 14). This cell lineage conversion might be applicable to the treatment of OS. We and others have previously identified TRAF2- and NCK-interacting protein kinase (TNIK) as a protein interacting with the TCF4 transcriptional factor (15, 16). TNIK is essential for the transactivation of Wnt signal target genes, and its expression is associated with poor prognosis in patients with hepatocellular (17), colorectal (18), and pancreatic (19) cancers. Amplification of the *TNIK* gene is detectable in 7% of gastric cancers (20), and *TNIK* is reportedly one of several putative driver oncogenes (21). Its inhibition reduces the population of cells expressing CD44, CD133, CD166, CD24, and CD29 and abrogates colorectal cancer stemness (22). We recently revealed the feasibility of targeting TNIK in synovial sarcoma and lung cancer (23, 24). In the present study, therefore, we investigated the potential of TNIK-targeted treatment for OS, revealing that TNIK is required for OS cell proliferation and stemness and that its inhibition leads to conversion of OS cells to adipocytes.

## Results

### TNIK inhibition suppresses OS cell growth.

**Figure 1:**
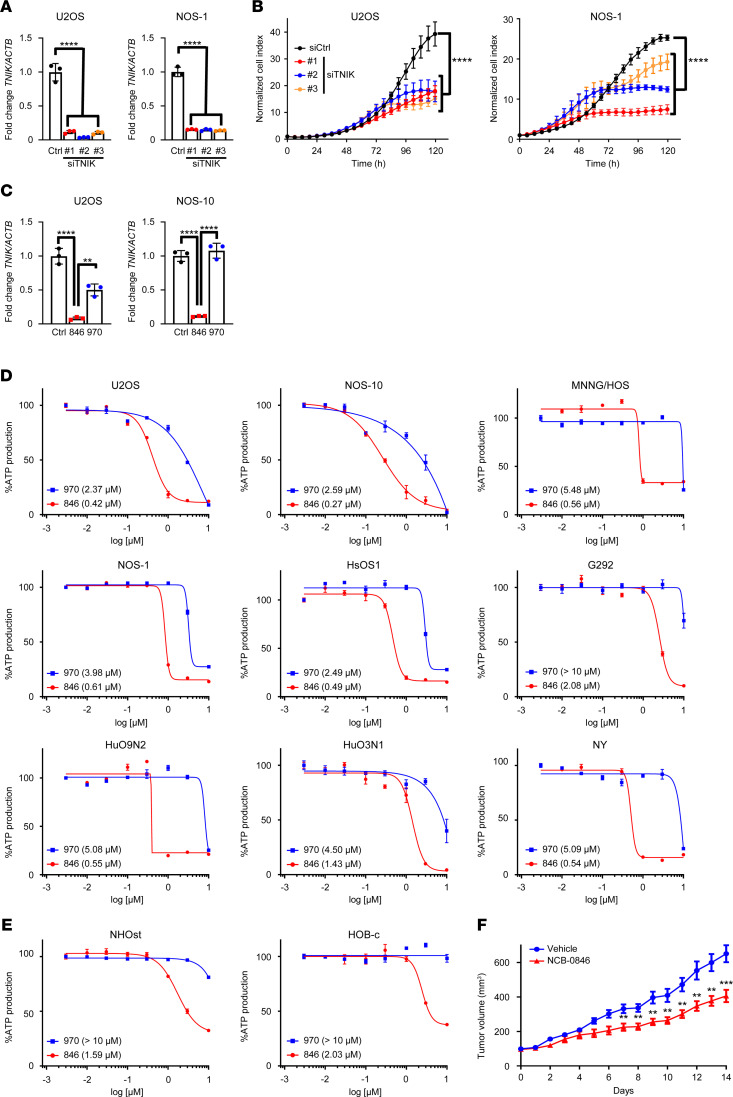
TNIK inhibition suppresses OS cell growth. (**A**) U2OS and NOS-1 OS cells were transfected with control siRNA (Ctrl) and siRNA against TNIK (siTNIK) in triplicate, and their expression of the *TNIK* gene (normalized to *ACTB*) was quantified by real-time RT-PCR. Data represent the mean ± SD of 3 replicates. One-way ANOVA, ****P < 0.0001. (**B**) Real-time growth monitoring of U2OS and NOS-1 OS cells transfected with control siRNA and siTNIK. Values at the time of transfection (0 hour) were set to 1. Data represent the mean ± SD of 4 replicates. One-way ANOVA, ****P < 0.0001. (**C**) The expression of *TNIK* (normalized to *ACTB*) in U2OS and NOS-10 cells treated with DMSO (Ctrl), NCB-0846 (846), or NCB-0970 (970) for 24 hours was quantified by RT-PCR. Data represent the mean ± SD of 3 replicates. One-way ANOVA, **P < 0.01, ****P < 0.0001. (**D**) Nine OS (U2OS, NOS-10, MNNG/HOS, NOS-1, HsOS1, G292, HuO9N2, HuO3N1, and NY) and (**E**) two osteoblast (NHOst and HOB-c) cell lines were cultured in the presence of 0.003–10 μM NCB-0846 or NCB-0970 for 72 hours, and their relative viability (IC_50_) was assessed in terms of ATP production. Data represent the mean ± SD of 3 replicates. (**F**) NOS-10 cells were inoculated into the subcutaneous tissues of 6-week-old female NOD/SCID mice. When the average volume of the xenografts reached 100 mm^3^, twice daily administration of water (vehicle, *n =* 10) or 80 mg/kg NCB-0846 hydrochloride (NCB-0846, *n =* 10) was initiated with a day off every 3 days and continued for a total of 15 days. Tumor growth and body weight in the vehicle group were compared with those in the NCB-0846 group at the same time points on a daily basis. Multiple *t* test corrected using the Holm-Sidak method, **P < 0.01, ***P < 0.001. Data represent the mean ± SEM.

To determine whether TNIK is a possible molecular target in OS, we analyzed the effects of *TNIK* gene silencing and a small-molecule TNIK inhibitor on OS cell proliferation. To ensure that the antiproliferative effects of siRNA constructs targeting *TNIK* (siTNIK) transfection in OS cell lines were due to selective depletion of TNIK, we used 2 additional siRNA constructs that targeted different regions of *TNIK* mRNA. Transfection of all 3 siTNIKs into U2OS and NOS-1 OS cells resulted in an up to 80% reduction of *TNIK* expression relative to cells transfected with control siRNA (Figure 1A). This *TNIK* gene silencing led to significant cell growth suppression (Figure 1B). A small-molecule TNIK inhibitor, NCB-0846 (22), suppressed the expression of *TNIK* (Figure 1C) and proliferation of 7 (U2OS, NOS-10, MNNG/HOS, NOS-1, HsOS, HuO9N2, and NY) of 9 OS cell lines examined with IC_50_ values of <1.0 μM (0.27–0.61 μM) (Figure 1D). However, the IC_50_ values for 2 osteoblast cell lines, NHOst and HOB-c, exceeded 1.0 μM (1.59 and 2.03 μM, respectively) (Figure 1E). To eliminate the possibility of off-target effects, we included NCB-0970, a diastereomer of NCB-0846 with a 13-fold lower level of TNIK-inhibitory activity (22). NCB-0970 hardly suppressed *TNIK* expression in NOS-10 cells but suppressed it by 50% in U2OS cells (Figure 1C). On the other hand, NCB-0846 reduced *TNIK* expression down to 8% in U2OS cells and 11% in NOS-10 cells. We further investigated possible mechanisms of inhibition of OS cell proliferation mediated by TNIK inhibition. NCB-0846 induced cleavage of poly (ADP-ribose) polymerase 1 (PARP-1) (Supplemental Figure 1A; supplemental material available online with this article; https://doi.org/10.1172/jci.insight.137245DS1) and an increase in the sub-G_1_ cell population (Supplemental Figure 1B), both of which indicate induction of apoptosis. To investigate whether NCB-0846 inhibits OS cell proliferation in vivo, we used a xenograft tumor model and confirmed that oral administration of NCB-0846 suppressed the growth of NOS-10 cells xenografted subcutaneously into immunodeficient mice and did not substantially affect their body weight (Figure 1F and Supplemental Figure 2). No apparent sign of toxicity was observed in the mice during the treatment period.

### RNA-Seq analysis of the effects of a small-molecule TNIK inhibitor in U2OS cells.

**Figure 2:**
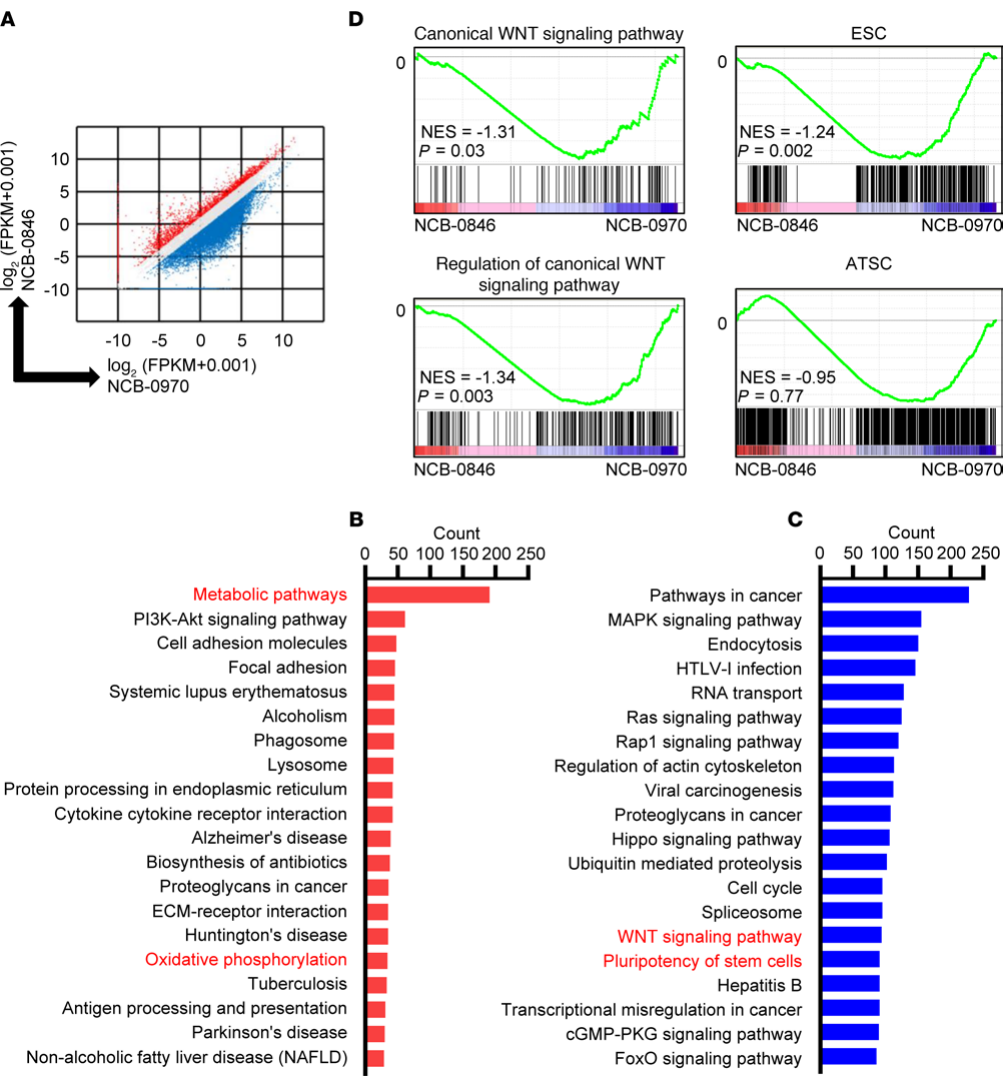
Transcriptome analysis of the effects of TNIK inhibitor in OS cells indicates upregulation of genes involved in metabolism and downregulation of genes involved in the Wnt signaling pathway and stem cell pluripotency. (**A**) Scatter plot analyses of differential gene expression in OS cells treated with NCB-0846 versus NCB-0970. U2OS cells were treated with 3 μM NCB-0846 or NCB-0970 for 24 hours. Each axis represents the log_2_ (FPKM + 0.001) value. Red dots indicate genes showing more than 2-fold upregulation, and blue dots indicate genes showing less than 0.5-fold downregulation. (**B**) Top 20 identified KEGG pathways from 3182 upregulated genes and (**C**) 11,920 downregulated genes. Counts of genes included in the pathways are depicted. (**D**) Gene set enrichment analysis (GSEA) showing significant enrichment of Wnt signaling–related genes (genes classified as being involved the canonical Wnt signaling pathway and its regulation) and an embryonic stem cell–related (ESC-related) gene set but not an adult tissue stem cell–related (ATSC-related) gene set, among genes downregulated by NCB-0846 (relative to NCB-0970) in U2OS cells. NES, normalized enrichment score.

We further performed transcriptome analysis to clarify the mechanisms behind the effects of a small-molecule TNIK inhibitor in OS cells. In general, NCB-0846 had a suppressive effect on transcription in OS cells (Figure 2A); it suppressed the expression of 11,920 (43%) of 27,339 transcripts compared with NCB-0970 in U2OS cells. Functional annotation of the RNA-Seq data (Supplemental Table 1) using the Database for Annotation, Visualization and Integrated Discovery (DAVID) bioinformatics resources (25) confirmed the substantial enrichment of pathways involved in metabolism among genes that were upregulated (>2-fold relative to NCB-0970) by NCB-0846 (Figure 2B), pathways involved in Wnt signaling, and the signaling pathway regulating the pluripotency of stem cells among genes that were downregulated (<0.5-fold relative to NCB-0970) by NCB-0846 (Figure 2C). NCB-0846 considerably suppressed the expression of genes involved in Wnt signaling (Supplemental Figure 3A and Supplemental Table 2) and the signaling pathway regulating the pluripotency of stem cells (Supplemental Figure 3B). A luciferase reporter assay revealed that NCB-0846 reduced the transcriptional activity of TCF/LEF in U2OS cells (Supplemental Figure 3C). Gene set enrichment analysis (GSEA) revealed that genes involved in Wnt signaling and functioning in embryonic stem cells (ESCs) were suppressed in NCB-0846–treated OS cells, whereas the adult tissue stem cell signature was not affected (Figure 2D). These results indicated abrogation of OS stemness by this small-molecule TNIK inhibitor, as we had observed previously in colorectal cancer (22).

### Metabolomic analysis of the effects of a small-molecule TNIK inhibitor in OS cells.

**Figure 3:**
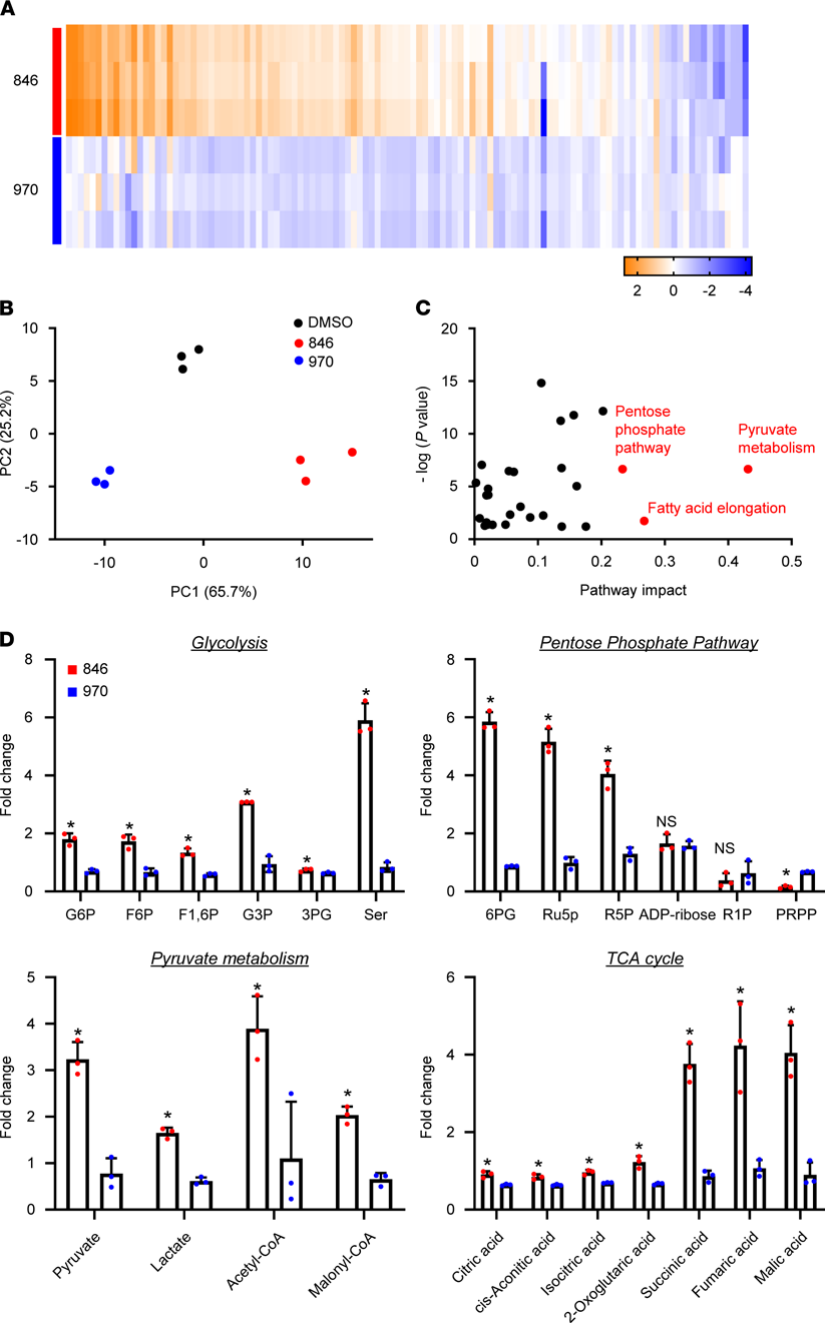
Metabolomic alterations associated with administration of TNIK inhibitor in OS cells. (**A**) Heatmap of 115 intracellular metabolites of U2OS cells treated with 1 μM NCB-0846 (846) or 1 μM NCB-0970 (970) for 72 hours. Relative concentrations or ratios of metabolites in U2OS cells treated with NCB-0846 or NCB-0970 were analyzed. The mean metabolite concentration in U2OS cells treated with DMSO was set as 1, and log_2_ values are depicted as a color scale (*n =* 3 per group). (**B**) Principal component analysis of metabolites of U2OS cells treated with DMSO (vehicle) (black), 1 μM NCB-0846 (red), or 1 μM NCB-0970 (blue) for 72 hours. (**C**) Pathway analysis showing high-impact alterations of the pentose phosphate, pyruvate metabolism, and fatty acid elongation pathways by NCB-0846. (**D**) Relative concentrations of metabolites involved in glycolysis, the pentose phosphate pathway, pyruvate metabolism/lipid synthesis, and the TCA cycle in U2OS cells treated in triplicate with 1 μM NCB-0846 (red) or 1 μM NCB-0970 (blue) for 72 hours. The mean metabolite concentration in U2OS cells treated with DMSO was set as 1. The complete metabolome data are available in Supplemental Table 3. L/P, lactate/pyruvate. Welch’s *t* test, *P < 0.05 compared with NCB-0970.

As RNA-Seq analysis implied metabolic activation of OS cells by pharmacological inhibition of TNIK, we investigated the metabolic state of OS cells treated with NCB-0846. The metabolic state of OS cells after NCB-0846 treatment was distinct from that of cells treated with NCB-0970 or DMSO (Figure 3, A and B; Supplemental Figure 3, A and B; and Supplemental Table 3). Metabolites involved in glycolysis, the pentose phosphate pathway, pyruvate metabolism/lipid synthesis, fatty acid elongation, and the tricarboxylic acid (TCA) cycle were markedly altered in OS cells (Figure 3, C and D. Increased cellular concentrations of malonyl-CoA and acetyl-CoA (Figure 3D) suggested that production of lipids was enhanced. This was thought to be due partly to increased production of pyruvate relative to the production of lactate (Figure 3D). Consistently, the expression of genes encoding rate-limiting enzymes for the production of lactate from pyruvate — lactate dehydrogenases (*LDHA*, *LDHC*) and pyruvate dehydrogenase kinases (*PDK2*, *PDK4*) — was markedly suppressed by NCB-0846 (Supplemental Figure 4A). Tumor cells generally exhibit aerobic glycolysis, but NCB-0846 induced a distinct metabolic change in OS cells that favored oxidative phosphorylation, which ultimately fuels lipid biosynthesis (Supplemental Figure 4B).

### Abrogation of OS stemness by TNIK inhibition.

**Figure 4:**
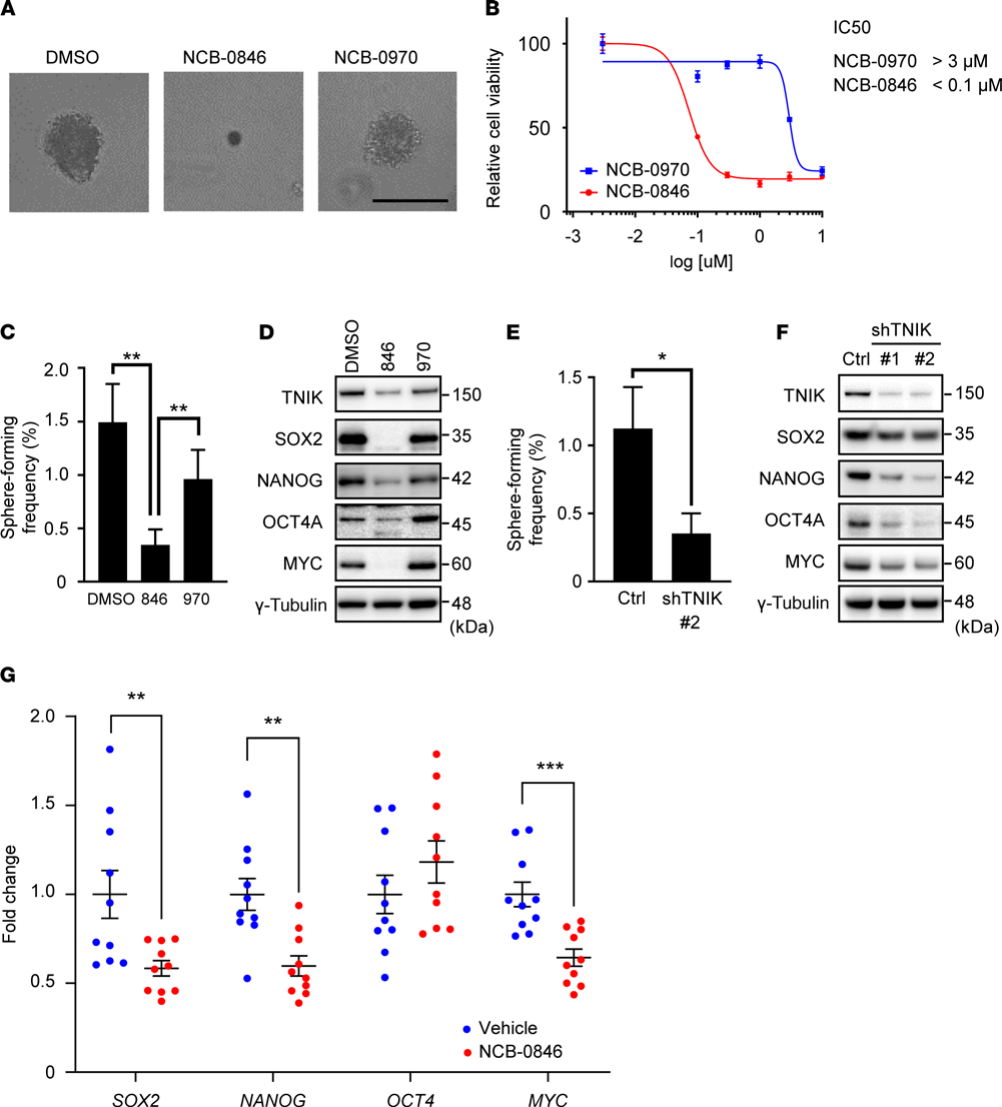
TNIK inhibition abrogates OS stemness. (**A** and **B**) Anchorage-independent colony formation by OS cells. U2OS cells were embedded in soft agar; covered by media containing DMSO alone (control), 1 μM NCB-0846, or 1 μM NCB-0970; and cultured for 14 days. (**A**) Representative images of colonies are shown (scale bar: 100 μm). (**B**) Relative cell viability was determined colorimetrically at 450 nm (*n =* 4). Data represent the mean ± SD. (**C**) Frequency of sphere formation by OS cells. U2OS cells were treated with DMSO, 1 μM NCB-0846, or 1 μM NCB-0970 for 3 days. The frequency of sphere formation and statistical significance were calculated using Extreme Limiting Dilution Analysis (ELDA) software. **P < 0.01. (**D**) Immunoblot analysis of TNIK and transcription factors related to cancer stemness (SOX2, NANOG, OCT4A, and MYC) in U2OS cells treated with DMSO, 1 μM NCB-0846, or 1 μM NCB-0970 for 72 hours. (**E**) TNIK knockdown suppresses the sphere-forming activity of OS cells. USO2 cells stably expressing shCtrl (Ctrl) or shTNIK (#2) were distributed into 96-well U-bottomed culture clusters at a density of 1, 10, or 100 cells per well and cultured for 14 days. The frequency of sphere formation and statistical significance were calculated using the ELDA software. *P < 0.05. (**F**) Immunoblot analysis of TNIK, proteins related to cancer stemness (SOX2, NANOG, OCT4A, and MYC), and γ-tubulin (loading control) in U2OS cells stably expressing shCtrl or shTNIK clone 33 (#1) and clone 35 (#2). (**G**) Expression of the *SOX2*, *NANOG*, *OCT4*, and *MYC* genes (normalized to *ACTB*) in NOS-10 xenografts excised from immunodeficient mice administered water (vehicle, *n =* 10) or 80 mg/kg NCB-0846 HCl (*n =* 10). Student’s *t* test, **P < 0.01, ***P < 0.001. Data represent the mean ± SEM.

One of our previous studies has demonstrated that TNIK inhibition abrogated colorectal cancer stemness (22), and the RNA-Seq analysis described above indicated that NCB-0846 downregulated genes involved in OS stemness, thus confirming loss of the cancer stem phenotype. NCB-0846 inhibited colony formation by U2OS cells in soft agar (Figure 4, A and B), and the IC_50_ value of NCB-0846 was calculated to be <0.1 μM in this anchorage-independent condition (Figure 4B). Limiting dilution assay showed that NCB-0846 and NCB-0970 reduced the frequency of sphere formation down to 23% and 64% of that of the DMSO control, respectively **(**Figure 4C). These assays were performed under 3-dimensional anchorage-independent culture and used for assessment of CSC function. The expression of transcription factors characteristic of ESCs, including *SOX2*, *NANOG*, *OCT4A*, and *MYC*, was decreased in U2OS cells after treatment with NCB-0846 but not with NCB-0970 (Figure 4D). To confirm that the effects of NCB-0846 on OS stemness were specifically the result of TNIK inhibition, we used small hairpin RNA (shRNA) targeting *TNIK* (shTNIK) and demonstrated that this had similar inhibitory effects on sphere-forming activity (Figure 4E) and expression of the NANOG, OCT4A, and MYC proteins in U2OS cells (Figure 4F). ALDH activity, which also reflects cancer stemness, was decreased by NCB-0846 (Supplemental Figure 5A) but not considerably suppressed by shTNIK (Supplemental Figure 5B). These transcription factors are the known targets of Wnt signaling (26–29) and are related to not only cancer stemness, but also the pathogenesis of OS (30, 31). Real-time reverse transcription–PCR (RT-PCR) confirmed the downregulation (<0.5-fold) of a large proportion (48 of 73) of known human Wnt target genes (Supplemental Table 2). Decreases in the expression of *SOX2*, *NANOG*, and *MYC* were also confirmed in tissues from mice treated with NCB-0846 (Figure 4G).

### PPARγ activation and conversion of OS cells to adipocytes after TNIK inhibition.

**Figure 5:**
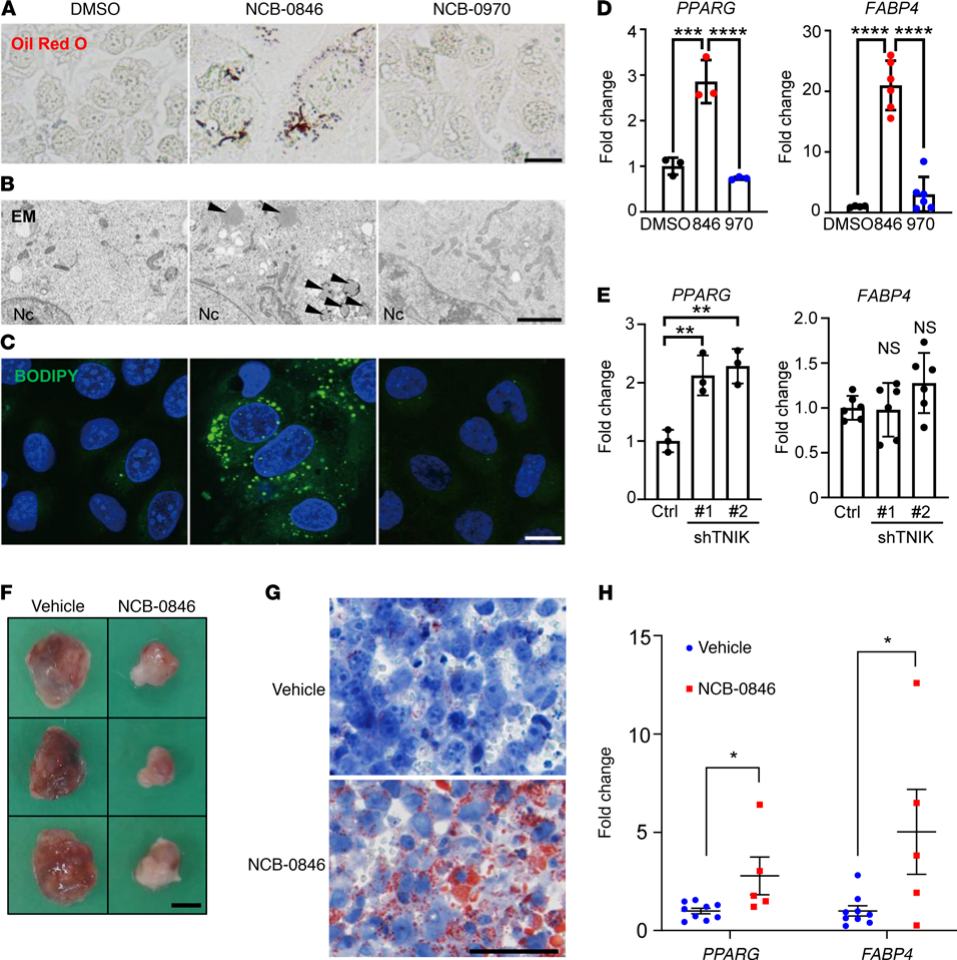
Conversion of OS cells to adipocyte-like cells through TNIK inhibition. (**A**–**C**) U2OS cells were treated with DMSO, 1 μM NCB-0846, or 1 μM NCB-0970 for 3 days, and intracellular lipid droplets were visualized by (**A**) Oil red O staining, (**B**) electron microscopy (EM), and (**C**) fluorescent molecular probe BODIPY labeling. Nc, nucleus. Arrowheads indicate lipid droplets. Scale bar: 20 μm (**A** and **C**); 3 μm (**B**). (**D**) U2OS cells were treated with DMSO, 1 μM NCB-0846, or 1 μM NCB-0970 for 3 days, and expression of the *PPARG* (*n =* 3, respectively) and *FABP4* (*n =* 4 for DMSO and *n =* 6 for NCB-0846 and NCB-0970) genes (normalized to *ACTB*) was quantified by RT-PCR. Data represent the mean ± SD. One-way ANOVA, **P < 0.01, ****P < 0.0001. (**E**) Expression of the *PPARG* (*n =* 3) and *FABP4* (*n =* 6) genes (normalized to *ACTB*) in U2OS cells stably expressing control shRNA or shTNIK was quantified by RT-PCR. Data represent the mean ± SD. One-way ANOVA, **P < 0.01. (**F**) Representative images of the macroscopic appearance of NOS-10 xenografts resected from mice administered vehicle or NCB-0846 HCl (90 mg/kg) for 10 days. Scale bar: 5 mm. (**G**) Oil red O staining for NOS-10 xenografts resected from mice administered vehicle or NCB-0846 HCl (90 mg/kg) for 10 days. Scale bar: 50 μm. (**H**) Expression of the *PPARG* and *FABP4* genes (normalized to *ACTB*) in NOS-10 xenografts resected from mice administered vehicle (*n =* 9) or NCB-0846 HCl (*n =* 5) for 10 days. Student’s *t* test, *P < 0.05. Data represent the mean ± SEM.

Activation of Wnt signaling is reported to suppress the adipogenesis of preadipocytes, and disruption of Wnt signaling causes transdifferentiation of myoblasts into adipocytes (14). We speculated that this phenomenon might also occur in OS cells. NCB-0846 treatment resulted in marked accumulation of lipid droplets positive for Oil red O staining in the cytoplasm of U2OS cells (Figure 5A) without the use of adipocytic differentiation-inducing cocktails (dexamethasone, methylisobutylxanthine, insulin, and troglitazone). We also confirmed this intracellular lipid droplet formation using electron microscopy (Figure 5B). Staining with the BODIPY 493/503 green fluorescent molecular probe demonstrated cellular accumulation of neutral lipid droplets (Figure 5C). The expression of *PPARG*, a master transcription factor of adipogenesis (32), and its target gene *FABP4*, a marker of adipogenesis, was increased by NCB-0846 (Figure 5D). Treatment with vehicle (DMSO) or NCB-0970 did not induce such lipid accumulation or expression of the adipocytic differentiation marker (Figures 5, A–D). Cells expressing shTNIK also exhibited higher expression of the *PPARG* gene (Figure 5E) PPARγ protein (Supplemental Figure 6A), confirming that NCB-0846 had driven the conversion of OS cells to an adipocyte fate by PPARG activation via TNIK inhibition. NCB-0846 and shTNIK increased the peroxisome proliferator response element-driven luciferase (PPRE-Luc) activity of U2OS cells (Supplemental Figure 6, B and C). The increased expression of a panel of key genes by NCB-0846 involved in the differentiation and maintenance of adipocytes (such as *GATA2/3*, ref. 33, and *FGF10*, ref. 34) was confirmed by real-time RT-PCR (Supplemental Table 4), but the expression of other adipogenesis induction genes, including *FABP4* (Figure 5E), was not suppressed in cells expressing shTNIK. OS xenografts resected from mice administered NCB-0846 had a white lipomatous appearance macroscopically (Figure 5F). Oil red O staining confirmed the in vivo adipocytic phenotype (Figure 5G). In addition, increased expression of *PPARG* and *FABP4* mRNA was confirmed in xenografts from the NCB-0846–administered mice (Figure 5H).

### Active TNIK expression in OS cells.

**Figure 6:**
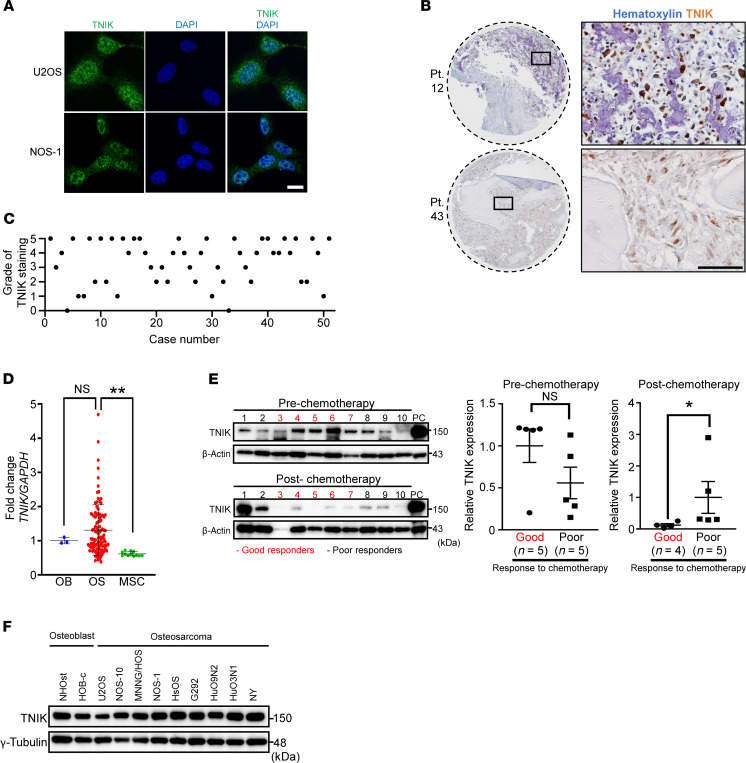
TNIK is highly expressed in the nuclei of human OS. (**A**) Immunofluorescence microscopy of TNIK (green) in U2OS cells. Nuclei (blue) were stained with DAPI. Scale bar: 20 μm. (**B**) Representative images of human OS tissues stained with anti-TNIK antibody. Rectangles indicate areas of magnification. Scale bar: 100 μm. (**C**) The 51 OS tissue samples were graded from 0 (no nuclear staining) to 5 (>75% of viable tumor cells) according to the percentage of tumor cells with nuclear TNIK expression. (**D**) Gene expression of *TNIK* (normalized to *GAPDH*) retrieved and analyzed from human OS data sets (19) in osteoblast (OB, *n =* 3), osteosarcoma (OS, *n =* 103), and mesenchymal stem cells (MSC, *n =* 12). The mean value of OB was set as 1. One-way ANOVA, **P < 0.01. (**E**) Blot intensity of TNIK (normalized to β-actin [loading control]) in the specimens before and after chemotherapy from good and poor responders. The sample after chemotherapy from case no. 3 was excluded from the analysis. Data represent the mean ± SEM. Mann-Whitney *U* test, *P < 0.05. PC, positive loading control. (**F**) Immunoblot analysis of TNIK and γ-tubulin (loading control) protein expression in osteoblast and OS cell lines.

To identify the status of TNIK for the clinical application of TNIK inhibitor as a potentially novel OS treatment, we performed immunostaining of TNIK in OS cell lines and tissue samples. Upon autophosphorylation, TNIK is translocated from the cytoplasm to the nucleus (35), and this nuclear localization is indicative of its active status. Immunofluorescence microscopy confirmed that TNIK was localized mainly in the nucleus of U2OS and NOS-1 cells (Figure 6A). TNIK was also detected in nuclei in the OS tissue samples examined (Figure 6B). Most of these OS tissue samples showed TNIK protein expression in the nuclei (Supplemental Figure 7) with varying degrees of positivity (grade 0 [no nuclear staining] to grade 5 [>75% of viable tumor cells]) (Figure 6C and Supplemental Table 5). Next, to determine whether OS specimens show any changes in *TNIK* mRNA relative to mesenchymal lineage cells, we analyzed data on gene expression deposited in the Gene Expression Omnibus (GEO) data set (GSE42352) (36). The level of expression of *TNIK* mRNA normalized to that of *GAPDH* mRNA was significantly increased in OS tissues relative to osteoblasts or MSCs (Figure 6D and Supplemental Data file 1). Additionally, we compared the expression of TNIK protein between OS tissue samples obtained by diagnostic incisional biopsy before neoadjuvant chemotherapy and those resected surgically after neoadjuvant chemotherapy. To avoid potential biases, 10 teenage patients in whom the primary tumor was located in the extremities and had no metastatic lesion at initial diagnosis were enrolled. TNIK was expressed in all specimens before chemotherapy but was barely detectable in samples after chemotherapy, which were carefully collected from the viable portions of tumors that had shown a good response (37) (Figure 6E). The same protocol of combinational chemotherapy employing methotrexate, doxorubicin, and cisplatin was used in all patients (38). In samples after chemotherapy, expression of TNIK in the poor response group was significantly higher than that in the good response group (Figure 6E). There was no significant difference in the level of TNIK protein expression between osteoblast and OS cell lines (Figure 6F).

## Interaction between TNIK and PPARγ in OS cells.

**Figure 7:**
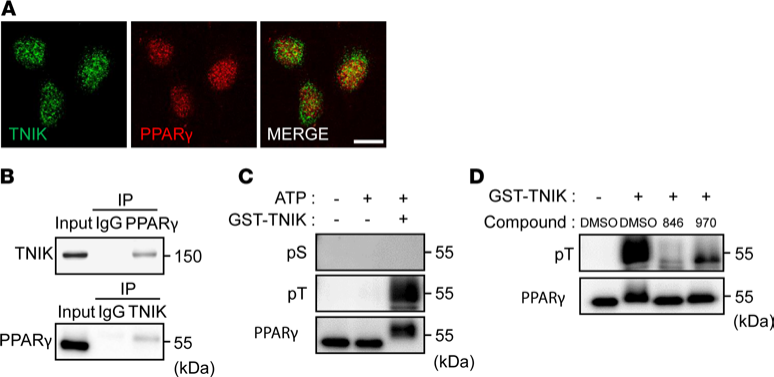
Interaction of TNIK with PPARγ. (**A**) Immunofluorescence microscopy of TNIK (green) and PPARγ (red) proteins in U2OS cells. Scale bar: 20 μm. (**B**) Interaction of TNIK with PPARγ. A nuclear protein extract of U2OS cells (input) was used for immunoprecipitation (IP) with anti-TNIK, anti-PPARγ, or relevant control IgG. Input and immunoprecipitated samples were immunoblotted with anti-TNIK and anti-PPARγ antibodies. (**C**) Threonine phosphorylation of PPARγ by TNIK. Full-length recombinant human PPARγ protein was incubated in the presence or absence of ATP and glutathione-S-transferase–tagged (GST-tagged) TNIK protein before immunoblotting with anti-phosphoserine (pS), anti-phosphothreonine (pT), and anti-PPARγ antibodies. (**D**) Full-length recombinant human PPARγ protein was incubated with GST-tagged TNIK protein in the presence of DMSO (vehicle), NCB-0846, or NCB-0970 before immunoblotting with anti-phosphothreonine (pT) and anti-PPARγ antibodies.

Finally, we analyzed the direct relationship between TNIK and PPARγ in U2OS cells. Immunofluorescence microscopy revealed colocalization of the TNIK and PPARγ proteins in the nuclei of U2OS cells (Figure 7A), and immunoprecipitation confirmed the interaction of TNIK and PPARγ in these cells (Figure 7B). As modulation of PPARγ activity by phosphorylation has been widely accepted, we further analyzed the phosphorylated forms of PPARγ (39–41). In vitro kinase assay revealed that TNIK directly phosphorylated threonine (but not serine) residue(s) of PPARγ (Figure 7C), and this phosphorylation was blocked by NCB-0846 (Figure 7D).

## Discussion

In the present study, we demonstrated for the first time to our knowledge the feasibility of targeting TNIK in OS. RNA interference or pharmacological inhibition of TNIK suppressed the growth and stemness of OS cells, converting them into adipocytes in vitro and in vivo. This potentially novel cell-lineage conversion coincided with the suppression of transcription factors essential for maintaining the ESC phenotype (SOX2, NANOG, OCT4, and MYC) and with the induction of the master regulator of adipogenesis, PPARγ. Additionally, active TNIK expression was confirmed in OS cells and samples of OS tissue from patients. These findings suggest that, by defining cell fate determination, TNIK would be a potential therapeutic target for OS.

An active TNIK state has been observed in the cell nuclei of several human cancers, including hepatocellular carcinoma, colon cancer, and leukemia, where it contributes to cell proliferation (17, 35, 42). Consistent with the tumorigenic role of TNIK, we found that TNIK inhibition suppressed the growth of OS cells. Expression of TNIK was higher in samples after chemotherapy from poor responders than in those from good responders. These preclinical data suggested that OS stem cells positive for TNIK had been intrinsically chemoresistant and preferentially selected by chemotherapy. Consistent with this finding, previous studies have suggested activation of canonical Wnt signaling in chemoresistant OS cells or OS stem cells (13, 43). Emerging data have indicated the involvement of Wnt signaling in the development and progression of OS (44). High-frequency canonical Wnt activation has also been observed in multiple sarcoma subtypes (45). Wnt signaling is reportedly activated in OS cells through aberration of various pathway components, including secreted frizzled-related protein-2 (46), Wnt inhibitory factor 1 (47), dickkopf-3 (48), and naked cuticle homolog-2 (49). The therapeutic potential of targeting some of these molecular components has been documented (44). However, no recurrent genetic alteration has been found in the Wnt pathway (4, 5), and no effective anti-Wnt therapeutic measures have yet been established (50). We have previously identified TNIK as an essential regulatory component of Wnt signaling in colorectal cancer cells (16). As TNIK was indispensable for the transactivation of Wnt signal target genes by TCF4 (15, 35), we screened a compound library and identified a small-molecule TNIK inhibitor, NCB-0846 (22). NCB-0846 suppressed the growth of colorectal cancer cells in vitro and in vivo through abrogation of colorectal cancer stemness. As is the case in colorectal cancer, here we also identified TNIK as a therapeutic target in OS, and pharmacological TNIK inhibition suppressed the growth of OS cells in vitro and in vivo. As we confirmed that the expression of TNIK was relatively higher in surgical specimens from poor responders compared with that in specimens from good responders, NCB-0846 in combination with standard OS chemotherapies may result in complete tumor regression, a possibility that warrants further investigation. As Wnt signaling is also known to be essential for maintenance of hematopoietic stem cells (51) and bone homeostasis (52), it would also be necessary to take preventive measures against anemia, leukopenia, or osteoporosis in patients receiving TNIK-targeted therapeutics for a long period of time.

Here we revealed that NCB-0846 had a significant inhibitory effect on the entire transcriptome (Figure 2A). *MYC*, *SOX2*, and *OCT4* are known target genes of Wnt signaling and are among the 4 factors originally used for reprogramming the genome and epigenome of adult somatic cells and producing induced pluripotent stem cells (53). *MYC* in particular is known to regulate as many as 10%–15% of genes in the genome, including many other transcription factors (54). It is anticipated that simultaneous suppression of these transcription factors would not only affect the expression of their own target genes, but also have a large effect on the entire transcriptome. In fact, we found that NCB-0846 suppressed 40% of human genes across the genome. This large-scale transcriptional reprogramming may be responsible for the cell-lineage conversion of OS.

Metabolomic analysis demonstrated that NCB-0846 had considerably positive effects on cellular metabolism in U2OS cells (Figure 3). Wnt signaling and its target genes have significant relationships to cellular metabolism (55, 56). Glycolysis, the pentose phosphate pathway, pyruvate metabolism, fatty acid elongation, and the TCA cycle were activated by NCB-0846 treatment. NCB-0846 led carbon flux toward lipid accumulation in OS cells as a result of increased acetyl-CoA production, which was well explained by decreased expression of lactate dehydrogenases and pyruvate dehydrogenase kinases. Previous studies have suggested that the levels of *LDH* and *PDK* expression are regulated by MYC (57, 58) and, therefore, that the intracellular metabolic changes induced by NCB-0846 may be the result of MYC inhibition. As MYC regulates all active promoters and enhancers in the cancer genome (59), it would be desirable to identify the key metabolic enzyme(s) responsible for the global metabolic alteration associated with OS development.

Abrogation of stemness and increased adipogenesis in OS cells resulting from TNIK inhibition were demonstrated using assays and molecular markers for stemness or adipogenesis in vitro and in vivo. OS is considered to be a disease in which osteoprogenitors fail to properly differentiate into bone-forming osteocytes, and, therefore, induction of terminal differentiation (into either osteocytes or adipocytes) would be a promising therapeutic approach. Recently, Takahashi et al. found that ROCK inhibition induces terminal adipocyte differentiation and confers chemoresistance in *MYC*-overexpressing OS cell lines (60). Basu-Roy et al. have demonstrated that thiazolidinediones, which act as PPARγ agonists, also promote growth arrest and adipogenic differentiation of OS cells (61). These results were consistent with our findings. To investigate the molecular mechanisms behind osteogenesis, adipogenesis, or stemness of mesenchymal cells, further details of the inverse relationship between the adipogenic and osteogenic lineage commitment of MSCs need to be clarified (62). Ross et al. have reported that Wnt inhibition by dominant-negative TCF4 causes adipogenesis and increases the expression of PPARγ in preadipocytes (14). Okamura et al. have also revealed that COUP-TFII acts downstream of Wnt signaling to silence PPARγ expression and repress adipogenesis (63). In addition, we have revealed here for the first time to our knowledge that TNIK directly interacts with PPARγ in U2OS cells and suppresses its activity, possibly by modulating the phosphorylation of threonine residues of PPARγ, which may provide a direct explanation for the inverse relationship between Wnt signaling and PPARγ activity.

There were certain limitations to our study. First, although NCB-0846 hydrochloride (HCl) can be given orally to mice without affecting their body weight, its safety and efficacy for long-term use should be assessed. Another limitation was the selectivity of NCB-0846 for TNIK, because NCB-0846 had an inhibitory profile with other kinases (22). For this reason, we used its diastereomer NCB-0970, siTNIK, and shTNIK, to minimize the likelihood of off-target effects. However, development of a TNIK inhibitor with higher selectivity will be needed to overcome this problem.

In summary, we have identified an active TNIK state in OS and demonstrated that TNIK inhibition suppressed tumor growth, abrogated OS stemness, and induced adipogenic conversion in vitro and in vivo. Together, our findings support the feasibility of TNIK as the first molecular target for OS treatment and may be of great value for further clinical development of targeted Wnt signaling in OS and multiple subtypes of sarcoma.

## Methods

### Cell lines.

Human OS NOS-10, HuO9N2, U2OS, HsOS1, NY, MNNG/HOS, NOS-1, HuO3N1, and G292 cells were obtained from the Health Science Research Resources Bank or Riken BioResource Center. Human osteoblast NHOst and HOB-c cells were obtained from Lonza and PromoCell, respectively. All the cell lines were maintained in media (listed in Supplemental Table 6) supplemented with 10% FCS (Thermo Fisher Scientific). Absence of mycoplasma contamination was routinely confirmed using the e-Myco VALiD Mycoplasma PCR Detection Kit (iNtRon Biotechnology). All cell lines were authenticated by the suppliers or by short tandem repeat profiling.

### Immunofluorescence microscopy.

Cells were fixed with 4% paraformaldehyde for 15 minutes and permeabilized in 0.5% Triton X-100 for 5 minutes. The fixed cells were incubated with a primary antibody overnight at 4°C and subsequently with a relevant secondary antibody (Alexa Fluor 488–conjugated anti-rabbit IgG or Alexa Fluor 546/555–conjugated anti-mouse IgG, Invitrogen) for 1 hour at 37°C. The nuclei were stained with DAPI (Vectashield HardSet Mounting Medium with DAPI, Vector Laboratories). Images were captured using a FluoView FV10i confocal microscope (Olympus).

### Immunohistochemistry.

A Human Osteosarcoma Tissue MicroArray (NBP2-30289) was purchased from Novus Biologicals. For antigen retrieval from formalin-fixed paraffin-embedded tissue sections, Target Retrieval Solution, pH 9.0 (S2367, Dako), or Liberate Antibody Binding solution (Polysciences Inc.) was used. Immunoperoxidase staining using the avidin-biotin complex was performed as described previously (64).

### Antibodies.

Antibodies used in this study are listed in Supplemental Table 7.

### Gene silencing by RNA interference.

Cells seeded at 50%–70% confluency were transfected with control siRNA (siCtrl) or siTNIK (s22905, s22906, s22907; Thermo Fisher Scientific) at a final concentration of 50 nM in accordance with the manufacturer’s instructions.

### Generation of stable cell lines.

Cells were transduced with lentivirus vectors expressing shTNIK (Mission Lentiviral Transduction Particle, clone 33 [#1, TRCN0000234733] and clone 35 [#2, TRCN0000234735], MilliporeSigma) or nontargeting control shRNA (MISSION Non-Target shRNA Control Transduction Particles [SHC002V], MilliporeSigma). The infected cells were selected with 2 μg/mL puromycin (Thermo Fisher Scientific) for 7 days and used for further analyses.

### Xenografts.

Five million NOS-10 OS cells suspended in PBS containing 25% Matrigel (BD Biosciences) were inoculated into the subcutaneous tissues of 6-week-old female NOD/SCID (NOD.CB17-Prkdcscid/J) mice (The Jackson Laboratory). When the average tumor volume reached approximately 100 mm^3^, the mice were randomized according to tumor volume and administered NCB-0846 HCl dissolved in water by oral gavage. Experiments were designed on the basis of at least 5 mice per treatment group, according to the results of our previous study (22). For the accurate comparisons of tumor size between treatments, we first inoculated NOS-10 cells into 40 mice. We excluded mice with an abnormal general status or mice that were judged to have poor tumor engraftment, and animals whose tumor volume was farthest from the mean on the day of grouping. We excluded a total of 10 animals in this manner and selected 30 animals, which were then divided into 3 groups (vehicle, low-dose NCB-0846 HCl, high-dose NCB-0846 HCl) of 10 using EXSUS (version 8.1, CAC Croit Co. Ltd.), ensuring that the tumor volumes and body weights of the mice on the day of grouping were almost equal. After starting treatment, the subcutaneous xenografted tumors were measured in a blinded manner. Data from the low-dose NCB-0846 HCl group were excluded because no effect on tumor volume was demonstrated. The original data are in the Supplemental Data file 2.

### Drug sensitivity assay.

Cells were seeded at a density of 3000 cells per well in 96-well plates. Twenty-four hours after seeding, the cells were exposed to serially diluted compounds (0.003, 0.01, 0.03, 0.1, 0.3, 1, 3, and 10 μM) and incubated for 72 hours. ATP production was measured using a CellTiter-Glo Luminescent Cell Viability Assay kit (Promega).

### Soft agar colony formation assay.

One milliliter 0.33% agar (Lonza) in culture medium containing 1.5 × 10^4^ cells was plated onto 1 ml solidified base agar (0.5%) in each well of 6-well clusters. The top agar layer was covered with culture medium containing serially diluted compounds. The medium was replaced every 3 days. Images were acquired after 14 days of incubation. Relative cell viability was determined colorimetrically at 450 nm using the Cell Count Reagent SF (Nacalai Tesque).

### Real-time cell analysis.

Cells were seeded at 5000 cells per well in 96-well clusters 1 day before transfection with siCtrl or siTNIK using LipofectAMINE RNAiMAX (Invitrogen). Cell growth was monitored periodically by a real-time cell electronic sensing analyzer (xCELLigence, ACEA Biosciences) for 120 hours via calculation of a cell index (normalized impedance) for each well. Each cell index was normalized to a set value of 1 at the time of siRNA transfection.

### RNA-Seq analysis.

Total RNAs were extracted from U2OS cells treated with DMSO, 3 μM NCB-0846, or 3 μM NCB-0970 for 24 hours. After confirming the absence of contamination with genomic DNA using a 2100 Bioanalyzer (Agilent), we used the TruSeq Stranded mRNA SamplePrep Kit to construct the sequencing library (Illumina), and the libraries were sequenced using Illumina HiSeq 2500 using a HiSeq PE Cluster Kit v4 and HiSeq SBS Kit v4-HS. Base calling was performed using Illumina Basecall Software (bcl2fastq v1.8.4) with default parameters.

Gene lists extracted from the transcriptome analyses were uploaded to the DAVID Bioinformatics database (https://david.ncifcrf.gov/), and the statistical significance of functional annotation was evaluated. Heatmaps were prepared from the genes included in the pathways related to this study and depicted using GraphPad Prism 8. GSEA software was used to evaluate the statistical significance of pathway enrichment and to calculate the normalized enrichment scores (http://software.broadinstitute.org/gsea/index.jsp).

### Real-time RT-PCR.

Total RNA was prepared using an RNeasy Plus Mini Kit (Qiagen) and purified with RNase-Free DNase (Qiagen) or with a Maxwell RSC simplyRNA Cells kit (Promega). cDNA was synthesized using a High-Capacity cDNA reverse transcription kit (Thermo Fisher Scientific) and subjected to TaqMan gene expression assay using predesigned primer and probe sets (listed in Supplemental Table 8). Amplification data measured as an increase in reporter fluorescence were collected using the StepOne Real-Time PCR System (Thermo Fisher Scientific). The relative mRNA expression level normalized to the internal control (human β-actin [*ACTB*] gene) was calculated by the comparative threshold cycle (C_T_) method (65). WNT Signaling Targets and Adipogenesis RT^2^ Profiler PCR Arrays (Qiagen) were used for pathway-focused gene expression analyses.

### Limiting dilution analysis.

One million U2OS cells were seeded onto 60 mm plates and treated with vehicle (DMSO) or 1 μM compound for 3 days. After washing off the compounds, the cells were dissociated to single cells in serum-free DMEM/F12 medium (Thermo Fisher Scientific) containing B27 (Thermo Fisher Scientific), 20 ng/ml epidermal growth factor (Thermo Fisher Scientific), 10 ng/ml basic fibroblast factor (Thermo Fisher Scientific), 5 μg/ml insulin (Thermo Fisher Scientific), 0.4% bovine serum albumin (MilliporeSigma), and 2 mM L-glutamine (Thermo Fisher Scientific). One hundred, ten, or one viable (determined by trypan blue dye exclusion) cells were seeded into each well of a 96-well U-bottom PrimeSurface plate (Sumitomo Bakelite). Two weeks later, the number of wells showing sphere formation was counted. The frequency of sphere-forming cells was calculated using Extreme Limiting Dilution Analysis (ELDA) software (http://bioinf.wehi.edu.au/software/elda/index.html) provided by the Walter and Eliza Hall Institute (22).

### Metabolomics.

Metabolome analysis was conducted using the C-SCOPE package of Human Metabolome Technologies (HMT), employing capillary electrophoresis time-of-flight mass spectrometry (CE-TOFMS) for cation analysis and CE-tandem mass spectrometry (CE-MS/MS) for anion analysis based on the methods described previously (66, 67). Briefly, CE-TOFMS analysis was carried out using an Agilent CE capillary electrophoresis system equipped with an Agilent 6210 time-of-flight mass spectrometer. The systems were controlled by Agilent G2201AA ChemStation software version B.03.01 for CE and connected by a fused silica capillary (50 μm i.d. × 80 cm total length) with commercial electrophoresis buffer (H3301-1001 and I3302-1023 for cation and anion analyses, respectively, HMT) as the electrolyte. The spectrometer was scanned from 50 to 1000 *m/z*. Peaks were extracted using MasterHands, automatic integration software (Keio University) (68) and MassHunter Quantitative Analysis B.04.00 (Agilent Technologies) in order to obtain peak information including *m/z*, peak area, and migration time (MT). Signal peaks were annotated according to the HMT metabolite database on the basis of their *m/z* values with the MTs. Concentrations of metabolites were calculated by normalizing the peak area of each metabolite with respect to the area of the internal standard and by using standard curves with 3-point calibrations. Acquired concentrations of metabolites were normalized to total protein contents and corrected using ATP concentrations.

Heatmap drawing and principal component analysis (PCA) were performed using Prism 8 (GraphPad) and XLSTAT (Addinsoft) software, respectively. A web-based metabolomic data processing tool, MetaboAnalyst 3.0 (69) (https://www.metaboanalyst.ca/), was used to calculate and depict the *P* and impact values of altered metabolic pathways.

### Oil red O staining.

Detection of intracellular lipid droplets was performed as described previously (70). In brief, cells seeded at various conditions were washed twice with PBS and fixed with 10% formalin at room temperature for 10 minutes. Fixed cells were washed twice with PBS and treated with 60% isopropanol for 1 minute. Filtered Oil red O staining solution was then added for 10–20 minutes at room temperature. After staining, the cells were washed once with 60% isopropanol and twice with PBS for further observation.

### Electron microscopy.

Samples were fixed with 2% paraformaldehyde and 2% glutaraldehyde (GA) in 0.1 M phosphate buffer (PB), pH 7.4, at incubation temperature and then kept at 4°C for 30 minutes followed by fixation with 2% GA in 0.1 M PB at 4°C overnight. The fixed samples were then dehydrated and embedded in resin (Quetol-812; Nisshin EM). Polymerized resins were ultrathin-sectioned at 70 nm with a diamond knife and mounted on copper grids. The mounted materials were stained with 2% uranyl acetate at room temperature for 15 minutes and then washed with distilled water followed by secondary staining with lead citrate solution (MilliporeSigma) at room temperature for 3 minutes. Digital images were obtained using a transmission electron microscope (JEM-1400Plus; JEOL Ltd.) and a charge-coupled device camera (EM-14830 RUBY2; JEOL Ltd.).

### Fluorescent molecular probe staining.

Neutral lipids were stained with 4,4-difluoro-1,3,5,7,8-pentamethyl-4-bora-3a,4a-diaza-s-indacene (BODIPY 493/503) and inspected by fluorescence microscopy (71).

### Immunoprecipitation.

Nuclear fractions of U2OS cells were prepared using NE-PER Nuclear and Cytoplasmic Extraction Reagents (Thermo Fisher Scientific). The lysates were incubated at 4°C overnight with the indicated antibody or relevant control IgG and precipitated with Dynabeads protein G (Dynal Biotech).

### Luciferase reporter assay.

Relative luminescence units for detecting the transcriptional activity of TCF/LEF were measured and calculated as reported previously (22, 35). For the PPAR reporter assay, a Cignal PPAR Reporter kit (CCS-3026L, Qiagen) was used in accordance with the manufacturer’s instructions.

### Patient samples.

Fresh frozen tissue samples were obtained from 10 teenage patients with OS with primary tumors in their extremities and no distant metastasis at the time of diagnosis before and after neoadjuvant chemotherapy at the National Cancer Center Hospital. Tumor samples were collected immediately after diagnostic incisional biopsy (before chemotherapy) or surgical resection (after chemotherapy) and cryopreserved in liquid nitrogen until use. The diagnosis of OS was confirmed by critically reexamining the clinical and histopathological findings.

### Data and materials availability.

The raw RNA sequence data have been deposited in the Sequence Read Archive database of the National Center for Biotechnology Information (SRP174040).

### Statistics.

All statistical analyses were performed using GraphPad Prism 8. Unless otherwise indicated, all cell-based experiments were run at least in triplicate, with at least 2 independent experiments, and 2-tailed Student’s *t* tests for 2 samples assuming equal variances were used to calculate the *P* values. Welch’s *t* tests (2 tailed) were used for analyzing metabolite concentration. Multiple *t* test (2 tailed) corrected using the Holm-Sidak method was used for comparing tumor volume in mice. For cell-based experiments with more than 3 groups, 1-way ANOVA was performed. Mann-Whitney *U* test was used for analyzing protein expression derived from OS clinical samples. Differences at *P* < 0.05 were considered significant.

### Study approval.

All the animal experiment protocols in this study were reviewed and approved by the institutional ethics and recombination safety committees of the National Cancer Center Research Institute. The minimum number of animals necessary for obtaining reliable results was used, and maximum attention was paid to animal rights and welfare protection. The use of human materials was reviewed and approved by the Institutional Review Board of the National Cancer Center. Written informed consent was obtained from each participant.

## Author contributions

TH, M. Masuda, and TY designed the research. TH, M. Masuda, T. Sugano, T. Sekita, NG, TA, T. Sakagami, YU, and NA performed the research. TH, M. Masuda, MS, and TY analyzed the data. All authors interpreted the data. M. Masuda, HM, MS, NA, MN, M. Matsumoto, RN, TK, AK, EK, and TY provided administrative, technical, or material support. TH, M. Masuda, and TY wrote the paper.

## Supplementary Material

Supplemental data

Supplemental Data Set 1

Supplemental Data Set 2

Supplemental Tables 1-8

## Version 1 01/05/2021

In-Press Preview

## Version 2 02/08/2021

Electronic publication
