# Supplementary material for: Direct conversion of osteosarcoma to adipocytes by targeting TNIK
Source: JCI Insight. 2021 Feb 8;6(3):e137245. doi: 10.1172/jci.insight.137245 (PMC7934882; doi:10.1172/jci.insight.137245)
Supplement: Supplemental data [file jciinsight-6-137245-s231.pdf]

Supplementary Figure S1

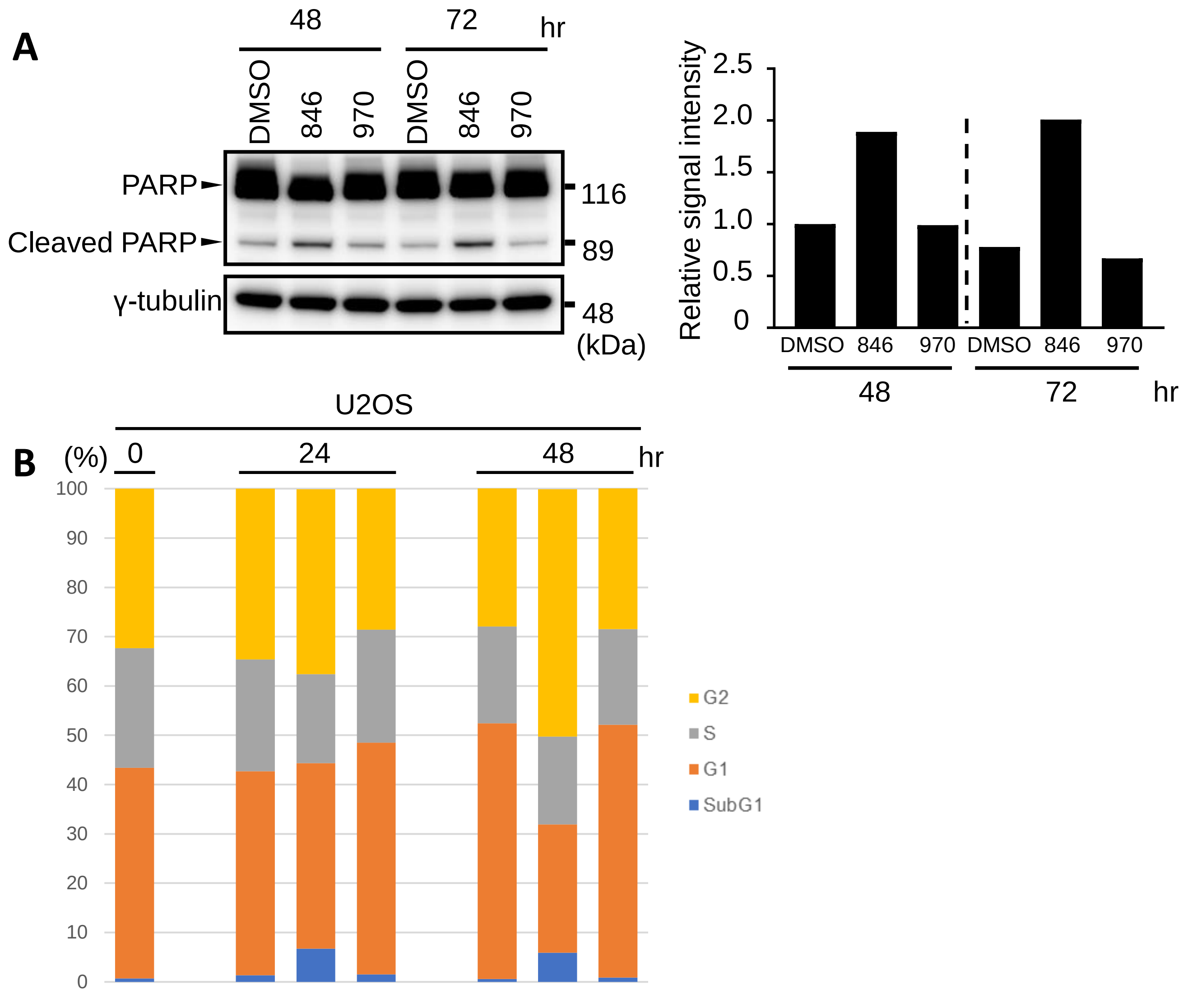

	Ctrl		DMSO	846	970		DMSO	846	970
Sub G1	0.7		1.3	6.7	1.5		0.6	5.9	0.9
G1	42.7		41.4	37.6	47.0		51.9	26.0	51.3
S	24.3		22.7	18.1	22.9		19.6	17.8	19.4
G2	32.3		34.6	37.5	28.6		28.0	50.2	28.5

Supplementary Figure S1. Induction of apoptosis in OS cells by NCB-0846.
(**A**) Immunoblot analysis of PARP-1, cleaved PARP-1, and γ-tubulin (loading control) proteins in U2OS cells treated with DMSO, 3 μM NCB-0846, or 3 μM NCB-0970 for 48 or 72 hours (left panel). The blot intensity of cleaved PARP-1 was quantified by densitometry (right panel). The intensity of cells treated with DMSO (vehicle) for 48 hours was set 1.
(**B**) U2OS cells were untreated (0) or treated with DMSO (vehicle), 1 μM NCB-0846, or 1 μM NCB-0970 for 24 or 48 hours, and the percentages of cells in the G2, S, G1, and subG1 phases were determined by flow cytometry.

Supplementary Figure S2

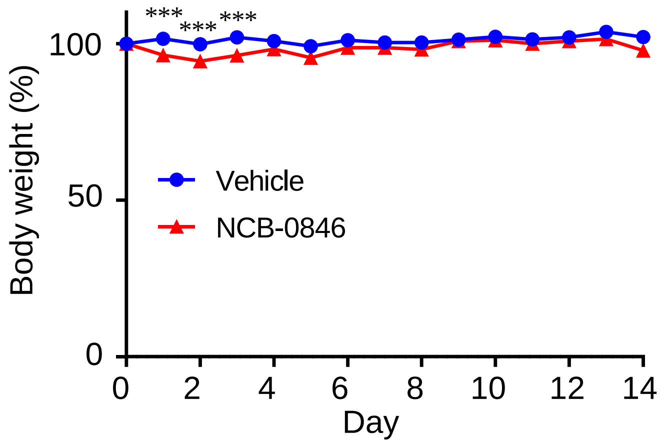

Supplementary Figure S2. The body weight of mice did not substantially affected by NCB-0846 administration.

NOS-10 cells were inoculated into the subcutaneous tissues of 6-week-old female NOD/SCID mice. When the average volume of the xenografts reached 100 mm3, twice daily administration of water (vehicle, *n* = 10) or 80 mg/kg NCB-0846 hydrochloride (NCB-0846, *n* = 10) was initiated with a day off every 3 days, and continued for a total of 15 days. Body weights were measured on a daily basis (Multiple t-test corrected using the Holm-Sidak method, ***P <0.001). Data represent the mean ± s.e.m..

Supplementary Figure S3

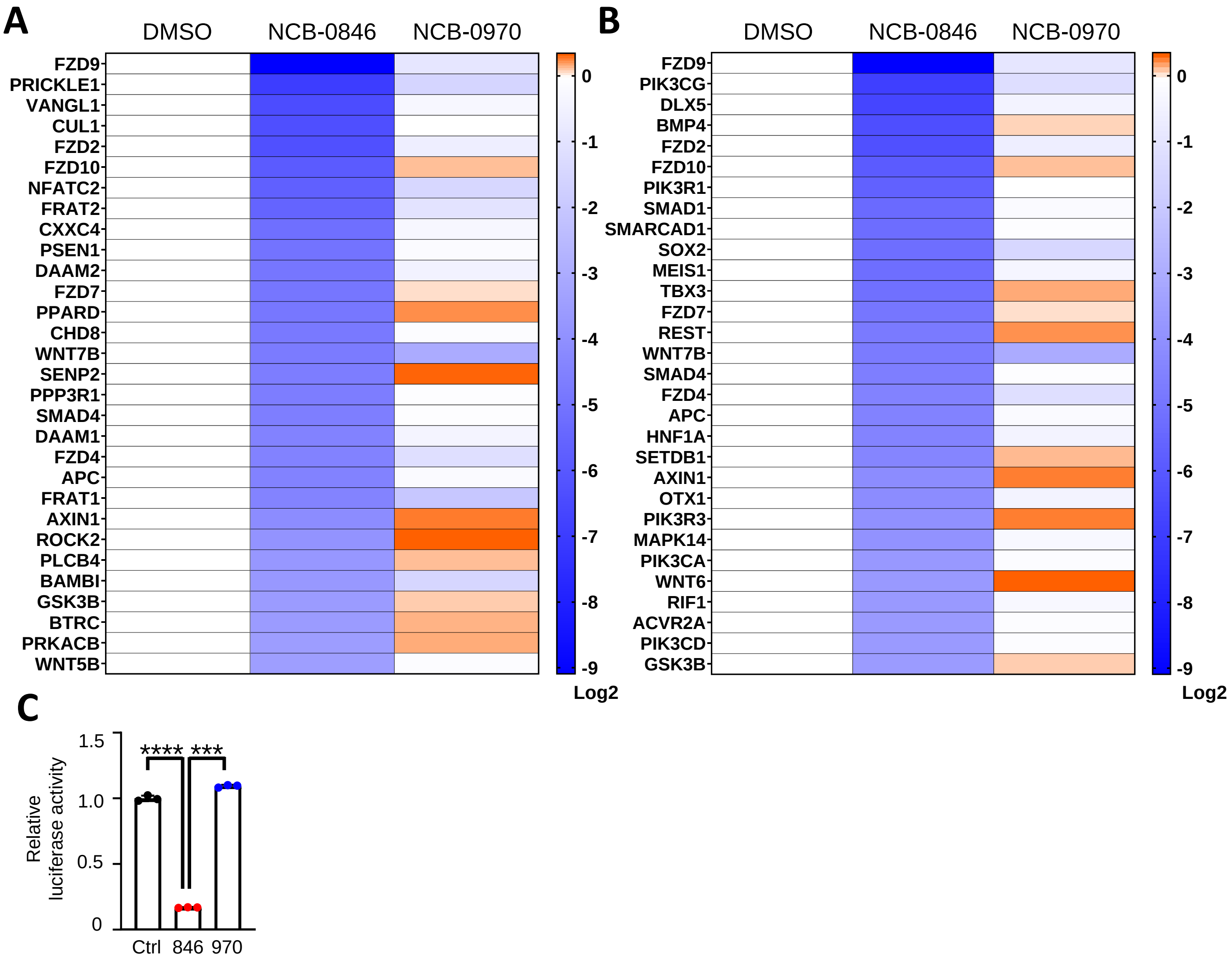

Supplemental Figure 3. NCB-0846 downregulates genes involved in the Wnt signaling pathway and genes involved in the pluripotency of stem cells.

U2OS cells were treated with DMSO (vehicle), 3 μM NCB-0846, or 3 μM NCB-0970 for 24 hours and subjected to RNA sequencing. Genes upregulated (orange) and downregulated (blue) by NCB-0846 and NCB-0970 are depicted as a color scale. We extracted the 30 top genes showing the largest variation between control (DMSO-treated) cells and NCB-0846-treated cells and created a heat map. The gene expression levels of U2OS cells treated with DMSO are set as 0 (white), and differences of log2 [Fragments Per Kilobase Million (FPKM) + 0.001] values are depicted as a color scale.

(**A**) Heatmap of genes classified into Wnt signaling pathway in the KEGG pathway database.

(**B**) Heatmap of genes classified into signaling pathways regulating the pluripotency of stem cells in the KEGG pathway database.

(**C**) Inhibition of TCF/LEF transcriptional activity in U2OS cells by NCB-0846. U2OS cells were transfected with the superTOPflash or superFOP-flash luciferase reporter in triplicate and treated with DMSO (Ctrl), NCB-0846 (846), or NCB-0970 (970). Data represent the mean ± s.d of 3 replicates. ***P <0.001, ****P <0.0001 (One-way ANOVA).

Supplementary Figure S4

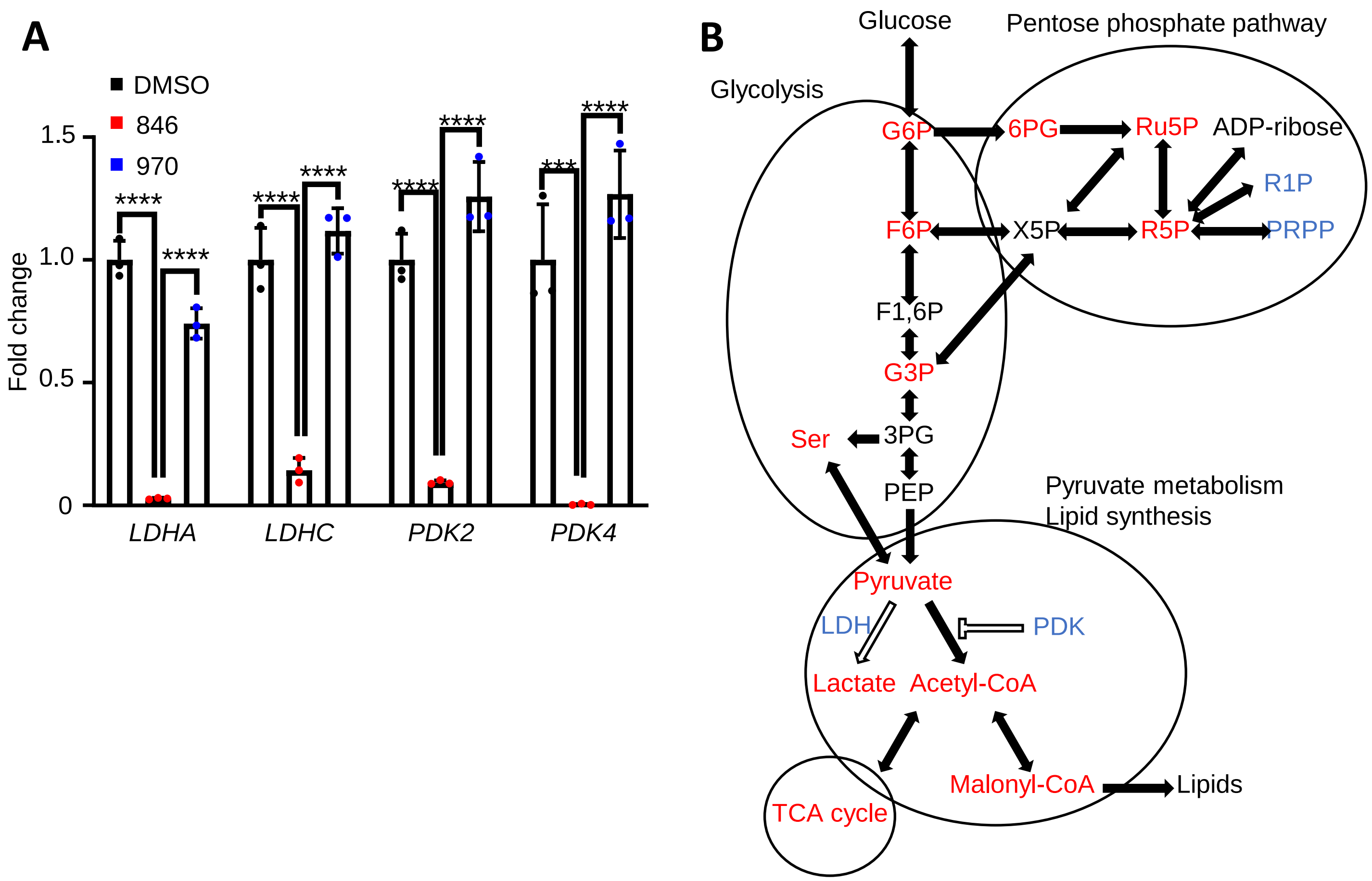

Supplementary Figure S4. Effects of NCB-0846 on the metabolic pathways and the expression of pyruvate/lactate metabolism-related genes.

(**A**) The relative expression of pyruvate/lactate metabolism-related genes (LDHA, LDHC, PDK2, and PDK4) in U2OS cells treated with DMSO (black), 3 µM NCB-0846 (red), or 3 µM NCB-0970 (blue) for 24 hours was quantified by real-time RT-PCR and normalized to ACTB. Data represent the mean ± s.d of 3 replicates. ***P <0.001, ****P <0.0001(One-way ANOVA).

(**B**) Schematic representation of the glycolysis, pentose phosphate, and pyruvate metabolism/lipid synthesis pathways with a link to the TCA cycle. Red and blue characters indicate metabolites whose concentrations increased and decreased, respectively, in U2OS cells treated with NCB-0846 in comparison to those treated with DMSO.

Supplementary Figure S5

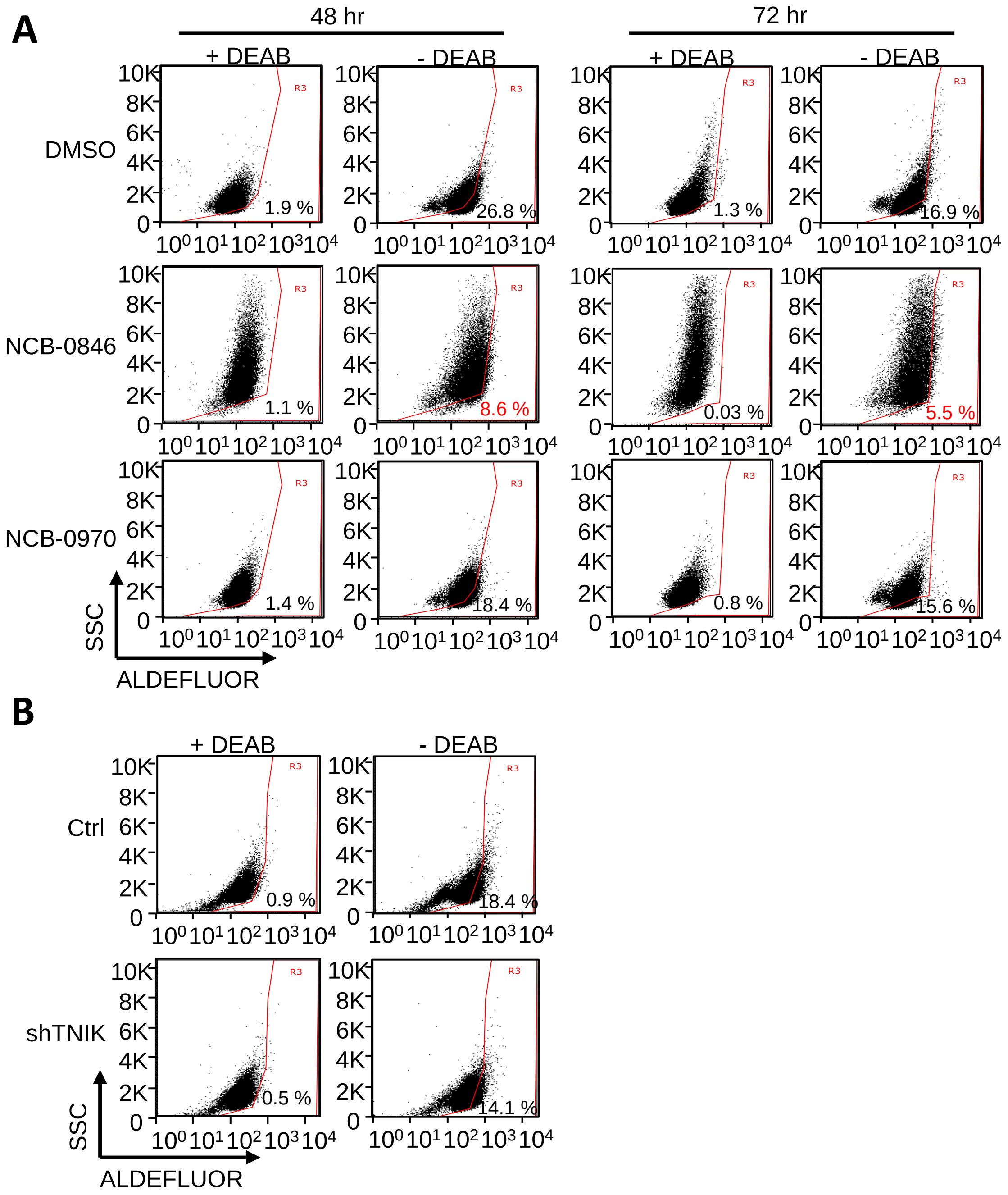

Supplementary Figure S5. Effects of NCB-0846 and shTNIK on ALDH activity in U2OS cells.
(**A**) ALDH activity in U2OS cells treated with DMSO, 1 µM NCB-0846, or 1 µM NCB-0970 for 48 or 72 hours. The population of cells with ALDEFLUOR™ intake in the presence (negative control) and absence of N,N-diethylaminobenzaldehyde (DEAB) was determined by flow cytometry. Gates were set according to the highest intake of ALDEFLUOR by cells in the DEAB control. Abbreviation: SSC, side scatter.
(**B**) ALDH activity of U2OS cells stably expressing shCtrl or shTNIK. The population of cells with ALDEFLUOR intake in the presence (negative control) and absence of DEAB was determined by flow cytometry. Gates were set according to the highest intake of ALDEFLUOR by cells in the DEAB control.

Supplementary Figure S6

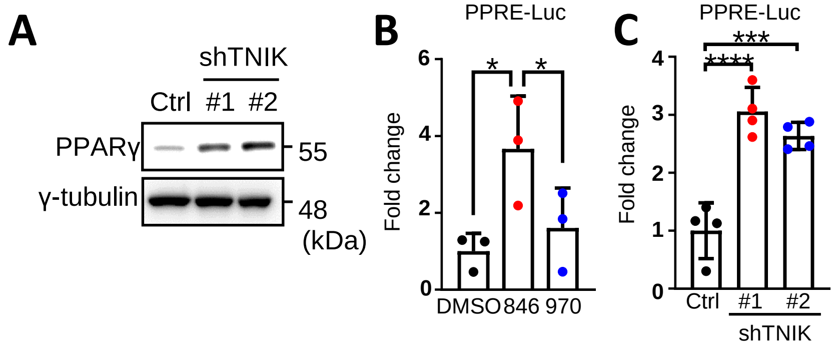

Supplementary Figure S6. TNIK inhibition activates PPARγ.

(**A**) Immunoblot analysis of the PPARγ and γ-tubulin proteins in U2OS cells stably expressing control shRNA or shTNIK.

(**B**) Peroxisome proliferator response element-driven luciferase (PPRE-Luc) activity of U2OS cells treated with DMSO, 1 μM NCB-0846, or 1 μM NCB-0970 for 3 days. Data represent mean ± s.d of 3 replicates. *P <0.05 (One-way ANOVA).

(**C**) PPRE-Luc activity of U2OS cells stably expressing control shRNA or shTNIK. Data represent mean ± s.d of 4 replicates. ***P <0.001, ****P <0.0001(One-way ANOVA).

Supplementary Figure S7

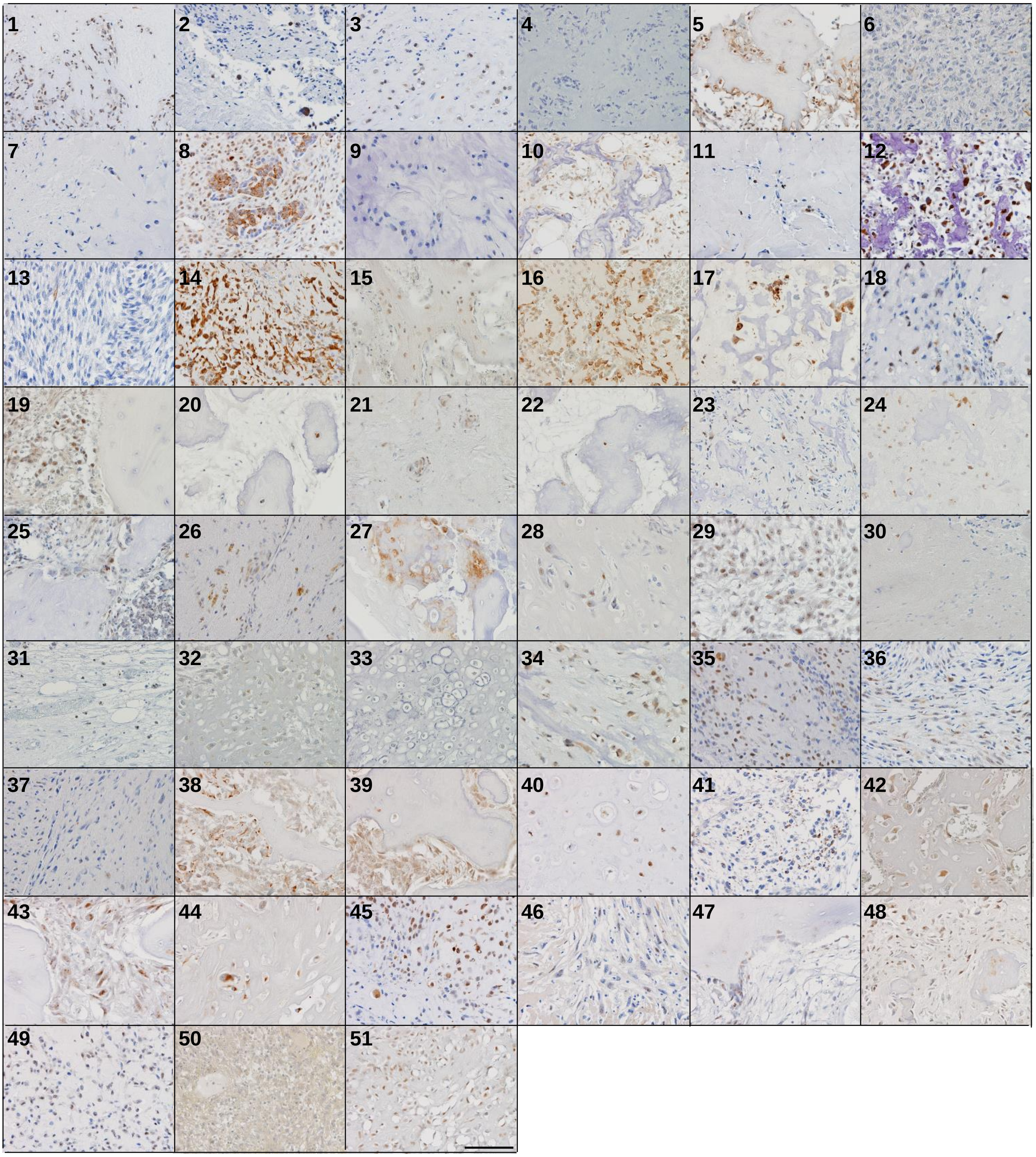

Supplementary Figure S7. Expression of TNIK in OS.
Tumor samples from 51 OS patients were stained with anti-TNIK antibody, and representative immunohistochemical images are shown. Images of #12 and #43 are shown in Figure 6B. Scale bar: 100 μm. Patient information is available in **Supplementary Table S5**.
